# Parkinson’s Disease and the Gut: Future Perspectives for Early Diagnosis

**DOI:** 10.3389/fnins.2020.00626

**Published:** 2020-06-17

**Authors:** Jana Harsanyiova, Tomas Buday, Alzbeta Kralova Trancikova

**Affiliations:** ^1^Departmet of Pahophysiology, Jessenius Faculty of Medicine in Martin, Comenius University, Bratislava, Slovakia; ^2^Biomedical Center Martin, Jessenius Faculty of Medicine in Martin, Comenius University, Bratislava, Slovakia

**Keywords:** Parkinson’s disease, alpha-synuclein, gastrointestinal tract, enteric nervous system, animal models, wholemount tissue staining

## Abstract

Parkinson’s disease (PD) is a neurodegenerative disease characterized by progressive degeneration of dopaminergic neurons, and at the cellular level by the formation of Lewy bodies in the central nervous system (CNS). However, the onset of the disease is believed to be localized to peripheral organs, particularly the gastrointestinal tract (GIT) and the olfactory bulb sooner before neuropathological changes occur in the CNS. Patients already in the pre-motor stage of PD suffer from various digestive problems and/or due to significant changes in the composition of the intestinal microbiome in this early stage of the disease. Detailed analyses of patient biopsies and autopsies as well as animal models of neuropathological changes characteristic of PD provided important information on the pathology or treatment of PD symptoms. However, presently is not clarified *(i) the specific tissue in the GIT* where the pathological processes associated with PD is initiated; (*ii) the mechanism* by which these processes are disseminated to the CNS or other tissues within the GIT; and (*iii)* which neuropathological changes could also serve as a *reliable diagnostic marker of the premotor stages of PD*, or (*iv) which type of GIT tissue* would be the most appropriate choice for routine examination of patient biopsies.

## Introduction

Parkinson’s disease (PD) with the prevalence of approximately 2%, is the second most common neurodegenerative disease in the world after Alzheimer’s disease (AD) (de Lau and Breteler, 2006), in which the incidence increases rapidly with age (Dauer and Przedborski, 2003). Most cases of PD, approximately 90%, represent the idiopathic forms of the disease (Hansen and Li, 2012). The disease is characterized by progressive degeneration of dopaminergic neurons in the central nervous system (CNS), namely, the *substantia nigra pars compacta (SNpc)*, and dopamine (DA) deficiency in the striatum, which is associated with the occurrence of many motor and non-motor symptoms (Dauer and Przedborski, 2003; Jankovic, 2008; Santos et al., 2019). In addition to the characteristic clinical motor symptoms such as tremor, bradykinesia, stiffness and postural instability, the disease is also manifested by non-motor symptoms, most commonly by dysfunction of the gastrointestinal tract (GIT) and olfaction (Braak et al., 2003).

**FIGURE 1:**
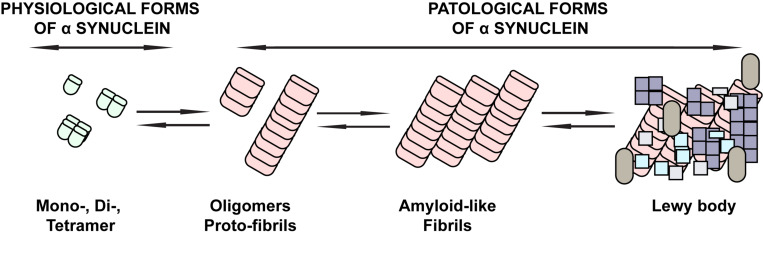
Schematic overview of the physiological and pathological forms of αS. The physiological forms of αS commonly found in the CNS or peripheral tissues of the CNS are mainly stable unfolded monomers or folded tetramers. Under pathological conditions, conformational changes of αS with beta-sheet formation occur that are associated with the occurrence of oligomers, followed by protofibrils and fibrils typically phosphorylated on serine 129, until they eventually deposit in Lewy bodies. These forms are considered to be pathological conformers of αS associated with the pathogenesis of neurodegenerative diseases.

The neuropathological feature of PD is the formation of intraneuronal cytoplasmic eosinophilic protein inclusions, Lewy bodies (LB) and Lewy neurites (LNs) in neurons of both the central and enteric nervous systems (ENS). The major component of LB and LNs, among other proteins, is pathologically aggregated alpha-synuclein (αS) (Braak et al., 2006; Caputi and Giron, 2018). The αS is a presynaptic protein whose exact physiological function is currently not fully understood, but its localization in the presynaptic nerve terminals may indicate an influence on the regulation of synaptic vesicle transduction (Cabin et al., 2002; Trancikova et al., 2011). Physiologically, αS most commonly occurs in the form of stable unfolded monomers (Eliezer et al., 2001) or folded tetramers (Bartels et al., 2011). Oligomers, protofibrils, and fibrils (Lashuel et al., 2013), typically phosphorylated at serine 129 (S129) (Okochi et al., 2000; Fujiwara et al., 2002) are considered to be pathological conformers associated with the αS pathogenesis in neurodegenerative diseases (Figure 1).

As early as the beginning of the 21st century, Braak et al. (2003, 2006) suggested that the development of PD initiates in the ENS based on the effect of an unknown toxin and/or pathogen, and progresses to the CNS through anatomical connections of these systems or that ENS may be affected by PD pathology in the initial stage of the disease. However, the exact GIT localization of initial PD pathology and specific mechanisms of disease spreading to the CNS are still not fully understood.

The aim of this article is to: (i) summarize experimental possibilities of early PD diagnosis based on biopsies and autopsies of PD patients and animal models; (ii) on the basis of current knowledge, delineate possible mechanisms by which the disease can develop and spread in the CNS, and (iii) finally, we focused on the complexity and lack of information in the studies and attempted to explore the possibilities for improving the detection of PD-related pathological processes in GIT tissues.

## Impact of PD-Associated Pathological Processes on the Enteric Nervous System

The enteric nervous system (“second brain”) is a complex neural network made up of about 100 million neurons (Furness, 2008) consisting of two plexuses: myenteric (Auerbach’s) and submucosal (Meissner’s). These innervate both the proximal part of GIT, the oral cavity, the salivary glands, the esophagus, and the stomach; as well as the distal GIT, the small intestine, colon and rectum (Lebouvier et al., 2009; Visanji et al., 2014). The myenteric plexus is located between the longitudinal and circular muscle layers throughout the entire GIT, where it controls mainly smooth muscle activity and motility. The submucosal plexus is mainly present in the small and large intestine, to a lesser extent in the stomach, while in the esophagus it is not present at all (Visanji et al., 2014). Enteric neurons are unevenly distributed in both plexuses, ENS along the entire GIT consists of intrinsic innervation located in the gastrointestinal wall and extrinsic innervation derived from sympathetic and parasympathetic nervous systems. Regulation of GIT function, such as motility and secretion by the ENS is independent of the CNS. However, ENS is bidirectionally connected to the CNS and together they form the so-called gut–brain axis (GBA) at the level of the autonomic nervous system (Lebouvier et al., 2009; Carabotti et al., 2015). Despite the fact that the ENS controls the physiological processes taking place in GIT independently of the CNS, the link between the two systems mediates their mutual influence (Rao and Gershon, 2016). Because of this connection, a healthy organism maintains intestinal homeostasis, and vice versa, neurodegenerative CNS diseases are reflected in ENS disorders and thus in gastrointestinal problems of patients. This causality is also seen in other neurodegenerative diseases such as AD, transmissible spongiform encephalopathies, or amyotrophic lateral sclerosis (Spielman et al., 2018), while GIT disorders often result in CNS-related symptoms (Caputi and Giron, 2018). The ENS, similar to the CNS, also contains a small proportion of dopaminergic neurons present at higher concentrations in both plexuses of the proximal parts of the GIT, with a decreasing tendency in its distal parts (Anlauf et al., 2003). However, the correlation between PD and the loss of dopaminergic neurons in GIT has only been demonstrated by Singaram in the analysis of colon tissues from patients with developed PD (Singaram et al., 1995). The study of the presence of LBs and LNs is still receiving a great deal of attention. We will deal with this issue in more detail in the following sections.

We must not forget the effect of the intestinal microbiome on the gut–brain relationship and their crosstalk. The intestinal microbiome constantly interacts with the ENS, either through bacterial metabolites or components of the bacterial walls and thus influences neural transmission in the ENS (Thomas et al., 2012; Forsythe and Kunze, 2013; Elfil et al., 2020). The intestinal epithelium forms a barrier that prevents the passage of harmful content and at the same time allows the absorption and secretion of nutrients (Marchiando et al., 2010; Chapelet et al., 2019) or drugs (Jin et al., 2019). The composition of the intestinal microbiota affects the proper function of this barrier, by regulating the expression of the tight junction proteins such as occludin, claudins, and zonula occludens-1 (ZO-1) (Bhattarai, 2018; Chapelet et al., 2019). Increased bowel permeability, called “leaky gut,” is typical for patients with early PD (Forsyth et al., 2011), when the function of the intestinal barrier is disrupted with subsequent inflammatory processes and increased oxidative stress. This environment further promotes αS accumulation and aggregation in the ENS (Sampson et al., 2016). Consistent with these findings, decreased expression of some tight junction proteins has been found in animal PD models (Gorecki et al., 2019; Dodiya et al., 2020) or in PD patients (Maes and Leunis, 2008; Clairembault et al., 2015a). In addition, increased expression of inflammatory cytokines and glial markers, also detected in GIT biopsies of PD patients, was positively correlated with disease progression and severity (Devos et al., 2013; Lin et al., 2019; Elfil et al., 2020).

An even higher incidence of peptic ulcer and *Helicobacter pylori* infections has been found in patients with PD up to 8–10 years before the onset of motor symptoms (Alvarez-Arellano and Maldonado-Bernal, 2014; Elfil et al., 2020). PD-associated neurodegeneration is likely to occur through the *Helicobacter pylori*-induced autoimmune and inflammatory response with increased production of pro-inflammatory cytokines and leukocyte activation. These can cross the disrupted blood–brain barrier (BBB), causing neurotoxicity and neurodegeneration (Dobbs et al., 2008; Elfil et al., 2020). In addition, *Helicobacter pylori* infection is associated with decreased L-DOPA absorption, whether due to *Helicobacter pylori*-induced inflammatory response in the gut of PD patients or increased gastric acid secretion. In these patients, eradication of *Helicobacter pylori* led to an improvement in L-DOPA responsive motor problems (Pierantozzi et al., 2006; Lee et al., 2008; Elfil et al., 2020). Several studies have confirmed reproducible and significant changes in the intestinal microbiome composition that correlate with PD-related motor symptoms (Scheperjans et al., 2015; Sampson et al., 2016; Perez-Pardo et al., 2017; Elfil et al., 2020). These changes are summarized in detail by several authors (Sun and Shen, 2018; Chapelet et al., 2019; Elfil et al., 2020; Shen, 2020).

These authors agree that the intestinal microbiome is also important for the development of the “parkinsonian intestine” phenotype. For example, a significantly increased concentration of *Enterobacteriaceae* in PD patients, which correlates with the severity of postural instability and gait difficulty. Conversely, reduced level of *Prevotellaceae* family correlates with increased intestinal permeability and vitamin B1 and B9 deficiency in these patients (Scheperjans et al., 2015). In addition, significantly reduced levels of the *Lactobacillaceae* family (Mukherjee et al., 2016) in patients with PD are mainly associated with the development of inflammatory processes, since anti-inflammatory effects are attributed to this bacterial family (Borre et al., 2014; Scheperjans et al., 2015; Unger et al., 2016). Decreased levels of some bacterial families (such as *Prevotellaceae, Lactobacillaceae, Lachnospiraceae*) in PD patients are interesting in terms of their ability to reduce short-chain fatty acid (SCFA) levels (Scher et al., 2013; Zmora et al., 2019) or ghrelin (Song et al., 2017; Yanagi et al., 2017). Both SCFA and ghrelin have a neuroprotective function, so their reduced levels observed in patients with PD can be considered not only as one of the critical factors of PD but also potentially reflect disease severity (Hill-Burns et al., 2017; Song et al., 2017; Elfil et al., 2020).

Intestinal microbiota may also affect drug metabolism and uptake, as confirmed by a negative correlation in the case of *Prevotellaceae* or, conversely, a positive correlation in the case of *Turicibacteraceae* (Lin et al., 2019). In this context, the positive effect of probiotic supportive treatment on the motor as well as non-motor symptoms of the disease was confirmed not only in PD patients (Tamtaji et al., 2019) but also in experimental animal models of PD (Srivastav et al., 2019; Hsieh et al., 2020).

## Localization of Pathological Forms of α-Synuclein in the ENS of PD Patients

Since Qualman et al. (1984) published the presence of LBs in the esophagus in 1 PD patient and 1 PD patient in the colon in 1984, an increasing number of post-mortem and *in vivo* studies have focused on finding or developing a suitable method for reliable detection of neuropathological changes in peripheral organs that would be suitable for taking biopsies. Pathologically aggregated αS in GIT has been identified to date in biopsies of PD patients from different parts of GIT (summarized in Table 1). However, the number of studies provided, the size of the individual groups used in these studies and controversy in obtained results (see below) does not make it possible to clearly determine the primary initiation site of the disease pathology onset within GIT and where or to which organs the pathology subsequently spreads (Cersosimo, 2015).

**TABLE 1:** Summary of studies focused on the presence of pathological forms of αS in various peripheral tissues.

Tissue	αS detected		αS form	Autopsy/biopsy	Number of patients and controls		Reference
Salivary glands	+	T	AGG	A	3	3	Cersosimo et al. (2011)
	+	P	LBs	B	15	0	Adler et al. (2014)
Pharynx	+	P	AGG	A	10	4	Mu et al. (2013)
Esophagus	ND	ND	LBs	A	22	50	Qualman et al. (1984)
	ND	ND	LBs	A	7	24	Wakabayashi et al. (1988)
	+	P	AGG	A	17	23	Beach et al. (2010)
	+	T, P	LBs	A	10	3	Gelpi et al. (2014)
Stomach	+	T	AGG	B	34	23	Sanchez-Ferro et al. (2015)
	+	T, P	LBs	A	10	0	Gelpi et al. (2014)
	+	P	LNs	B	1	0	Pouclet et al. (2012)
	+	P	ND	B	35	161	Hilton et al. (2014)
	+	T	ND	A	5	5	Braak et al. (2006)
Small intestine	+	P	LNs	B	1	0	Pouclet et al. (2012)
	+	P	ND	B	15	161	Hilton et al. (2014)
Appendix	+	T	ND	B	0	20	Gray et al. (2014)
Colon	+	P	LNs	B	5	8	Lebouvier et al. (2009)
	+	P	LNs	B	29	10	Lebouvier et al. (2010)
	+	T	AGG	B	9	23	Shannon et al. (2012a)
	+	T	ND	A	10	77	Gold et al. (2013)
	+	P	ND	B	62	161	Hilton et al. (2014)
	+	P	LNs	B	31	11	Clairembault et al. (2015a)
	+	T, P	ND	B	22	11	Visanji et al. (2015)
	+	T, P	ND	B	19	8	Antunes et al. (2016)
	+	T	LTS	B	9	3	Corbille et al. (2016)
	+	T, P	AGG	B	17	13	Corbille et al. (2017)
	+	P	AGG	B	18	11	Fenyi et al. (2019)

*The only direct evidence of PD-related neuropathological changes in the ENS seems to be the confirmation of the presence of pathological forms of αS and protein inclusions in the peripheral tissues of PD patients. The table summarizes the numbers of patients and controls in each study, broken down by the peripheral tissue of interest. Detected forms of αS and the study outcomes are indicated. PD, Parkinson’s disease; αS, α Synuclein; ND, not defined; T, total; P, phosphorylated at S129; AGG, aggregated; LBs, Lewy bodies; LNs, Lewy neurites; LTS, Lewy type synucleinopathy; A, autopsy; B, biopsy.*

### Salivary Glands and Pharynx

Salivary glands as a tissue with a relatively high number of αS aggregates have been studied in autopsies (Beach et al., 2010) as well as in biopsies of PD patients (Cersosimo et al., 2011). The presence of αS inclusions in minor salivary glands biopsies was confirmed in all PD (*n* = 3) patients, whereas control biopsies (*n* = 3) did not show this pathology (Cersosimo, 2015).

A needle core biopsy of the submandibular gland of PD revealed the presence of LBs was confirmed in 75% of cases but the study did not include control patients or patients with another type of neurodegenerative disease. However, due to minimal invasiveness, the possibility of using only local anesthesia, and the overall simplicity and reproducibility, this method presents a suitable method for monitoring Lewy body pathology in patients with PD (Adler et al., 2014).

Dysphagia is one of the many clinical problems of PD patients but often leads to more serious clinical complications, such as aspiration pneumonia, the most common cause of death in PD patients (Mu et al., 2013). Analysis of the pharynx autopsies of patients with confirmed PD (*n* = 10) and control samples (*n* = 4) confirmed the presence of phosphorylated αS (P- αS) aggregates in the sensory nerve axons of all PD patients with PD, compared to control samples. Patients with dysphagia even exhibited more αS-positive fibers than PD patients without diagnosed dysphagia. These results, therefore, suggest that pharyngeal sensory nerves are primarily affected by PD pathology, which may be related to subsequent sensitization of pharyngeal tissue, presence of oedema and further development of dysphagia in PD patients (Mu et al., 2013).

### Esophagus

At about the same time as Qualman published the first mention of LBs in ENS, Wakabayashi et al. (1988) focused on autopsies of GIT tissues in patients with histologically confirmed PD (*n* = 7). The presence of LBs was observed across the entire GIT with the highest concentration in the myenteric plexus at the lower part of the esophagus. However, healthy individuals (8/24) also showed LB-positivity (Wakabayashi et al., 1988). Extensive analysis of the presence of P-αS in autopsies of various tissues of patients not only with PD (*n* = 17) but also AD (*n* = 19), dementia with LBs (DLB, *n* = 9) and incidental Lewy body disease (*n* = 7) in comparison with healthy individuals (*n* = 23), confirmed the rostrocaudal P-αS distribution in the GIT of PD patients. The highest incidence was detected in the submandibular gland and lower part of the esophagus, while the concentration of aggregates decreased in the stomach, small intestine, colon, and rectum and even no presence of P-αS positive inclusions has been reported in the upper 2/3 of the esophagus (Beach et al., 2010).

Similarly, in a post-mortem histopathological study of tissues derived from the CNS-and autonomous nervous system from PD patients PD (*n* = 10), DLB (*n* = 5) and others (*n* = 13) including atypical parkinsonism and dementia without LBs (*n* = 13), the presence of αS aggregates was confirmed in all PD and DLB case, in contrast to the atypical form of parkinsonism and dementia without LBs. These aggregates have been confirmed not only in GIT tissues, but also in the adrenal gland, heart, sympathetic ganglia, vagus nerve, and genitourinary tract, with the typical rostrocaudal distribution. Distal esophagus and stomach contained the most observed αS aggregates, while the rectum contained the least (Gelpi et al., 2014).

### Stomach and Small Intestine

Analysis of the gastric mucosa in PD patients, control subjects, including cases with premotor symptoms, confirmed αS inclusions in the gastric mucosa were found in (17/28) patients with PD, in control biopsies (1/23) and patients with premotor symptoms (1/6) (Sanchez-Ferro et al., 2015). Besides that, the intraneuronal αS-positive inclusions have also been observed across the entire gastric ENS (cardia, fundus, pylorus), in the myenteric and submucosal plexus, as well as in the peripheral nerves found in the adventitia, in autopsies obtained from the brain and stomach of patients with sporadic PD (Braak et al., 2006). Also, the presence of αS-positive LNs was determined in fundic, antral and duodenal submucosa biopsies from one PD patient (Pouclet et al., 2012).

### Colon

To date, the colon has been the most studied part of the GIT in relation to PD (Schneider et al., 2016). Several studies have confirmed the occurrence of P-αS-positive LNs in colon biopsies from PD patients suggesting that conventional colon biopsies can be used to study the submucosal plexus of the ENS (Lebouvier et al., 2009, 2010; Shannon et al., 2012a, b; Gold et al., 2013; Visanji et al., 2014; Clairembault et al., 2015a).

In colon biopsies from PD patients (21/29) but not in healthy individuals (0/10), Lebouvier observed αS-positive LNs along with the loss of neurofilament-positive neurons in the ganglia. This increased number of LNs correlated with PD symptoms (Lebouvier et al., 2010). Similarly, Clairembault et al. (2015a) detected P-αS-positive LNs in the submucosal plexus of the colon (23/31). In all cases, control samples were negative for P-αS.

Several studies have focused on early PD symptoms in the GIT, and thus on pre-motor PD patients (Shannon et al., 2012a, b; Hilton et al., 2014; Visanji et al., 2014). Shannon et al. (2012a) confirmed the presence of αS in colon biopsies confirmed in all premotor PD patients (9/9) but not in healthy individuals (0/23), supporting the hypothesis of the incidence of pathological αS in GIT tissues in the pre-motor stages of the disease. In addition, increased oxidative stress was present in PD patients as well as in both control groups, suggesting that the pathological occurrence of αS is not the result of an inflammatory process or oxidative stress (Shannon et al., 2012b). Visanji et al. (2015) compared the presence of αS and P-αS in colon biopsies from patients at various stages of the disease (early PD *n* = 15; late PD *n* = 7) and healthy individuals (*n* = 11). However, the analysis did not confirm the difference in the presence of αS and P-αS within the studies groups, mainly due to the high positivity for αS and P-αS in control samples (Visanji et al., 2015). To date, the largest study has focused on analyzing the accumulation of pathological P-αS in various types of GIT tissues in patients with a very early pre-motor phase of the disease, at least 8 years before the first motor symptoms of PD. Together, they studied 117 biopsies from 62 PD patients and 161 samples from healthy subjects. Accumulation of P-αS was confirmed in mucosal and submucosal neurites in only seven PD patients, with all control biopsies without positive findings. Regarding GIT tissues, P-αS positivity was detected in gastric, duodenal and colon biopsies. However, in contrast to the abovementioned studies by Qualman et al. (1984), Wakabayashi et al. (1988), and Gelpi et al., 2014, esophageal samples did not confirm this positivity (Hilton et al., 2014).

In colon mucosa biopsies of idiopathic PD patients, αS positivity was present in most PD subjects studied (18/19), but P-αS was found in all PD patients (19/19) and all healthy individuals (8/8) (Antunes et al., 2016). When comparing colon biopsy samples of PD patients with AD and healthy individuals, up to 53% of all study subjects being positive for αS in an age-independent manner. While all samples from PD patients (10/10) were positive for αS, with significantly higher prevalence and expression compared to the control group, AD patients were in most cases negative for the presence of αS or did not exceed the control group (Gold et al., 2013).

A recent study focused not only on the detection of pathological forms of αS in the GIT, but the results have been correlated with non-motor symptoms of the disease (Lee et al., 2018). However, the presence of αS in gastric and colonic biopsies of PD patients (*n* = 35) and healthy individuals (*n* = 52) did not significantly correlate with complex GI dysfunction such as constipation, dyspepsia, abdominal pain or with the deterioration of a specific intestinal segment (Lee et al., 2018). Despite the relatively large sample of subjects studied, this study has several limitations: (i) analysis of deeper intestinal layers (especially ENS of submucosal and myenteric plexuses) and other GIT tissue types, (ii) immunohistochemical staining for pathological αS, (iii) the use of conventional histological approach only, (iv) lack of other experimental methods (e.g., biochemical) and (v) the low sample quality.

In general, the results agree that immunohistochemical confirmation of colorectal αS pathology in PD patients may be a suitable pre-mortem diagnosis of the disease. However, due to the slight inconsistency of the results in relation to healthy individuals, it is necessary to extend the standard immunological analysis to other types of analysis. Corbille et al. (2016) evaluated and compared four different immunohistochemical methods for optimal detection of Lewy-type synucleinopathy from submucosal colon biopsies of PD patients (*n* = 9) and healthy individuals (*n* = 3). However, none of the compared αS detection methods, granular staining in the *lamina propria*, perivascular/vascular wall submucosal staining, lacy-granular pattern in the submucosa and epithelial cell nuclear staining, showed specificity and sensitivity of more than 80%, which evaluates these methods as inadequate for routine PD diagnostics (Corbille et al., 2016). Similarly, biochemical analysis by one-dimensional or two-dimensional electrophoresis of colon biopsy samples from 17 PD patients (*n* = 17) and healthy individuals (*n* = 13) did not confirm a difference in αS expression levels, phosphorylation and aggregation between the groups in the study (Corbille et al., 2017). These findings reflect the limitations of individual methods suggesting the need for new and more sensitive methods, as well as the necessity to combine several methodologies for an accurate diagnosis.

Furthermore, Fenyi et al. (2019) recently studied GIT biopsies from the antrum, sigmoid colon and rectum from 18 PD patients and 11 healthy individuals. However, the classical immunohistochemical detection has been extended by the protein misfolding cyclic amplification (PMCA) assay. Interestingly, control subject with positive PMCA test subsequently developed PD symptoms. The immunohistochemical detection revealed the presence of P-aS in 5/15 PD samples, with four of these samples also being PMCA positive, indicating the diagnostic potential of the PMCA method. Control samples did not show any positivity for P-aS. Rectal biopsy does not represent a possibility of obtaining material in the case of PMCA due to insufficient number of aS aggregates (Fenyi et al., 2019).

### Appendix

It is now well documented that the vermiform appendix is part of the immune tissues associated with GIT, and also serves as a reservoir of the intestinal microbiome (Killinger and Labrie, 2019). Nevertheless, the mechanisms by which the appendix affects intestinal immunity and microbiome are still unknown today. In relation to PD, epidemiological studies have found that early appendectomy correlates with a reduction in the risk of developing PD (Killinger et al., 2018). This is probably due to the presence of aggregated aS also in healthy individuals in the mucosa and enteric plexuses of the appendix (Killinger et al., 2018). The fact, that a higher density of αS aggregates in the appendix was detected in PD patients as well as asymptomatic patients (Stokholm et al., 2016) suggest, that misfolded αS accumulates in the appendix, from where the pathology may have spread to other organs. Therefore, early removal of lymphoid organs with accumulated misfolded αS could potentially represent a therapeutic tool for patients in the early stages of PD or with familiar form (Gray et al., 2014; Killinger et al., 2018).

## Animal Models

Animal models in PD research represent an irreplaceable source of new knowledge, whether in connection with the understanding of the pathomechanisms that of PD initiation and progression, but also as preclinical models of PD (Metzger and Emborg, 2019). They can also help to elucidate whether intestinal dysbiosis is a cause or a consequence of PD-related pathology. However, no animal model can recapitulate all the symptoms and processes associated with the pathogenesis and progression of PD (summarized in Table 2) (Klingelhoefer and Reichmann, 2015). Just in an effort to address these shortcomings, a new generation of PD models is constantly being developed (Metzger and Emborg, 2019).

**TABLE 2:** Overview of the most used animal models of PD, their associated phenotypes and AS pathology.

Model	Type	Motor phenotype	Non-motor phenotype	Animal	αS/Lewy pathology
Toxin-induced	Rotenone	✓	✓	Mouse, rat	Yes
	MPTP	✓	✓	Mouse, primate	No
	6-OHDA	✓	✓	Rat	No
	Paraquat	✓	✓	Mouse	Yes
Genetic	αS overexpressing	✓	✓	Mouse	Yes
	αS-A53T transgenic	Not consistent	✓	Mouse	Yes
	Pink1	–	✓	Mouse	No
	MitoPark	✓	✓	Mouse	No
Propagation	αS PFF	✓	✓	Mouse	Yes

*αS, α Synuclein; MPTP, 1-methyl-4-phenyl-1,2,3,6-tetrahydropyridine, 6-OHDA, 6-hydroxydopamine, PFF, recombinant αS preformed fibrils.*

### Toxin-Induced Models

Toxin-induced models are widely used models mainly due to the relatively simple use of the toxin, clear phenotype, the rapid onset of motor or non-motor symptoms of the disease and the ability to reflect motor complications of antiparkinsonian drugs, such as L-DOPA-induced dyskinesia (Morin et al., 2014; Taguchi et al., 2020b). The disadvantage is the exposure of experimenters to toxins, the frequent criticism that the concentrations used to induce PD-related symptoms do not commonly occur in the natural environment, but in particular that robust motor phenotypes may interfere with the analysis of non-motor symptoms (Taguchi et al., 2020b).

### Rotenone Models

Rotenone is a highly lipophilic pesticide that readily crosses the BBB, spreads to neurons, and accumulates in mitochondria, where it acts as an inhibitor of mitochondrial complex I (Duty and Jenner, 2011). Rotenone-induced rodent models of PD are able to reproduce the neuropathological, anatomical as well as behavioral properties of the disease (Betarbet et al., 2000). In addition to the neurodegeneration of the nigrostriatal dopaminergic system and the presence of cytoplasmic inclusions in these neurons, accompanied by motor problems (Tieu, 2011), non-motor symptoms at the GIT level were also observed (Drolet et al., 2009; Greene et al., 2009; Tieu, 2011). However, Tasselli et al. (2013) did not confirm the delay in gastric emptying after oral application of rotenone to mice, despite the present neurodegeneration in *SNpc*. Similarly, the presence or absence of LB or pathological αS in the GIT or ENS depends on the route of rotenone administration, the dose used, and the length of exposure (Drolet et al., 2009; Greene et al., 2009; Pan-Montojo et al., 2010; Metzger and Emborg, 2019).

Regarding microbiota changes in the GIT, a significant decrease in the beneficial *Bifidobacterium* bacteria (Perez-Pardo et al., 2018) or, conversely, an increase in Gram-negative mucin-degrading bacteria (Akkermansia) (Dodiya et al., 2020) were observed in the rotenone-induced PD model. Chronic stress is another factor leading to an increase in intestinal hyperpermeability and a reduction of anti-inflammatory bacteria (*Lactobacillus*) in experimental animals. In combination with rotenone, chronic stress even promoted its potential to induce CNS pathology, manifested by a significant increase in the number of dystrophic microglia, increased reactivity of lipopolysaccharides (LPS) in *SNpc* and decreased DA in the striatum compared to control mice (Dodiya et al., 2020).

### MPTP Models

MPTP (1-methyl-4-phenyl-1,2,3,6-tetrahydropyridine) is a potent neurotoxin with high affinity for the DA transporter, as a result of which the metabolite MPTP (MPP+) accumulates in the mitochondria and acts as an inhibitor of mitochondrial complex I (Cleeter et al., 1992). MPTP is used to induce a parkinsonian-like phenotype in a number of mammalian animal models, e.g., in monkeys, mice, rats, swine, cats and sheep (Tieu, 2011). The mouse and monkey model are most commonly used, as the sensitivity to MPTP is comparatively lower in other mammals (e.g., rats) (Hisahara and Shimohama, 2010). The neuropathological effect of MPTP on the nigrostriatal DA pathway in animal models is similar to that observed in PD patients (Langston et al., 1999).

In ENS, an MPTP-induced significant decrease in dopaminergic neurons, tyrosine hydroxylase-positive (TH+), in the myenteric plexus has been described in monkeys (Chaumette et al., 2009) as well as in a mouse model of PD (Anderson et al., 2007). Other GIT dysfunctions have been described in a mouse model, but the information is controversial in some cases. While Anderson et al., 2007 reported increased MPTP-induced colon motility as well as unchanged emptying time compared to control animals, several other authors observed a significant reduction in colonic motility and constipation (Natale et al., 2010; Ellett et al., 2016). Selective loss of TH+ neurons was observed in the myenteric and submucosal plexuses of the colon, but not in the esophagus and stomach. The decrease in DA was accompanied by an increase in αS levels (Natale et al., 2010). Furthermore, in mice, MPTP-induced loss of TH+ neurons in ENS led to a strong immune response in the GIT (Cote et al., 2015; Ellett et al., 2016). Interestingly, partial depletion of pro-inflammatory M1 monocytes had a positive effect in terms of protecting ENS from TH+ neuronal loss, but this effect was ton manifested in the CNS (Cote et al., 2015). On the other hand, therapeutics (CuII) with a neuroprotective effect on the CNS improve not only motor functions but also intestinal functions defined by improved stool frequency, reduced enteric glial cell reactivity and markers of inflammation, as well as restoration of neuronal subpopulations in myenteric plexus damaged by MPTP exposure. These results suggest the existence of similar pathomechanisms in the CNS and ENS (Ellett et al., 2016).

### 6-OHDA Models

6-OHDA is a hydroxylated DA analog acting as a selective catecholaminergic neurotoxin that inhibits mitochondrial complex I and IV (Glinka and Youdim, 1995) and produces reactive oxygen species in cells (Cohen and Heikkila, 1974). Since 6-OHDA cannot effectively cross the BBB, direct injection of this toxin into a specific site in the brain is required (Perese et al., 1989; Przedborski et al., 1995; Hisahara and Shimohama, 2010; Duty and Jenner, 2011). As early as 1968 (Ungerstedt, 1968), complete anterograde degeneration of the nigrostriatal dopaminergic neural system associated with motor problems was described in rats after administration of 6-OHDA into the *SNpc*. In addition to the ability to recapitulate the motor symptoms of the disease (Hisahara and Shimohama, 2010; Tieu, 2011), the 6-OHDA rat model mimics many of the biochemical features of PD, such as a reduction in DA and TH levels in the striatum (Duty and Jenner, 2011). However, like many other animal models of PD, the 6-OHDA model fails to induce the formation of LBs, an important pathological feature of PD.

Regarding pre-motor symptoms, several authors reported PD-like constipation in 6-OHDA animal models (Blandini et al., 2009; Zhu et al., 2012; Levandis et al., 2015; Zhang et al., 2015; Fornai et al., 2016). Unilateral (Zhu et al., 2012) or bilateral (Zheng et al., 2011) injection of 6-OHDA to *SNpc* of rats leads to a general decrease of central DA as well as delayed gastric emptying, suggesting that central DA may regulate GIT function (Zheng et al., 2011; Zhu et al., 2012; Metzger and Emborg, 2019).

### Paraquat Models

The herbicide Paraquat, (1,1’-dimethyl-4,4’-bipyridine) is the inducer of αS aggregation and the oxidative stress in dopaminergic neurons (Kuter et al., 2007, 2010). Anselmi et al. (2017, 2018) reported a paraquat-induced decrease in gastric motility, and gastric tone, followed by increased αS immunoreactivity in dorsal motor nucleus of the vagus (DMV) in a rat model of PD (Anselmi et al., 2017, 2018). Co-administration of subthreshold doses of paraquat with lectin directly into the stomach of rats leads to a progressive PD-like phenotype, represented by the decrease in TH+ neurons in *SNpc*, the presence of misfolded αS in the *SNpc* and DMV, and L-DOPA responsive motor deficits. These symptoms were preceded by gastric dysmotility. Interestingly, vagotomy was able to block the spread of misfolded αS outside the myenteric neurons and even prevented the development of PD-related symptoms in these rats (Anselmi et al., 2018).

### Genetic Models

Since the number of patients with the familial form of PD is significantly lower than the incidence of an idiopathic form of the disease, genetic research of affected families is very demanding (Hisahara and Shimohama, 2010). Particularly because of this fact, it is extremely important to develop appropriate genetic animal models for research to investigate the etiopathology of PD. In these models, we must not forget that the choice of promoter fully influences the observed phenotype, whether in terms of the level of expression or the regions in which this gene will be expressed. A disadvantage compared to toxic models is the slower onset of the phenotype, and also that only a few models show loss of DA neurons and an associated motor phenotype (Taguchi et al., 2020b). Nevertheless, these models can significantly contribute not only to the understanding of PD-related pathomechanisms but also in the development of symptomatic or disease-modifying therapies that target non-motor symptoms associated with the disease. Therefore, efforts are constantly increasing to develop a PD model that would reproduce several PD-specific premotor symptoms at the same time, followed by slowly progressive DA neurodegeneration (Taguchi et al., 2020b).

### αS Overexpressing Models

In the case of αS, it is known that quantitative, as well as qualitative changes in αS, contribute to the development of PD. Several models have been developed, either based on over-expression of the wt αS gene (*SNCA*) or with the insertion of pathological mutations (A53T, A30P) (Singleton et al., 2003; Chartier-Harlin et al., 2004; Ibanez et al., 2004; Taguchi et al., 2020b). As mentioned above, the choice of strong exogenous promoters used in these models affects not only the phenotype but also the spatial and temporal distribution of the pathology, which may not always reflect the real human PD pathology. However, in general, we can say that αS transgenic models are able to recapitulate αS aggregation, similar to PD patients (Taguchi et al., 2020b).

Overexpression of αS (Thy1-αS) results in decreased basal stool, slower colonic transit time with significantly higher stool content (Wang et al., 2008). This manifestation was preceded by increased αS in myenteric plexuses with numerous varicose terminals surrounding specifically immunoreactive pChAT neurons (Wang et al., 2012). Sampson et al. (2016) revealed that in Thy1-αS mice, microbial depletion reduces the CNS pathology, characterized by reduced accumulation of pathological αS in the brain, as well as motor and GIT dysfunction. Microbiota transplants from PD patients lead to a significant deterioration of intestinal dysfunction as well as overall PD symptoms in these mice (Sampson et al., 2016). Similarly, colonization of curli (amyloid protein)-producing *Escherichia coli*, in Thy1-αS mice induces the αS pathology in both the gut and brain, along with classical intestinal and motor dysfunctions. Interestingly, treatment of these mice with a gut-restricted amyloid inhibitor blocks the progression of curli-induced pathology (Sampson et al., 2020). These results confirm the role of intestinal microbiota and bacteria-produced amyloids in the progression of PD-related CNS pathology, presumably through microglial activation and neuroinflammation, suggesting a potential positive effect of antibiotic treatment on ameliorating GIT as well as CNS symptoms in PD patients (Sampson et al., 2016, 2020).

Several studies have addressed the induction of the PD phenotype by lipopolysaccharide (LPS). LPS is a pro-inflammatory bowel-derived bacterial endotoxin that has been shown to induce progressive nigrostriatal pathology (Qin et al., 2007; Liu et al., 2008; Kelly et al., 2014). In addition, an increased amount of LPS-producing *Gammaproteobacteria* or mucin-degrading bacteria *Verrucomicrobiaceae* has been confirmed in patients with PD (Gorecki et al., 2019). In Thy1-αS mice, low dose oral administration of LPS induces the early onset of motor symptoms along with increased αS in the CNS. However, microbiota analysis did not confirm a higher rate of *Gammaproteobacteria* or the amount of *Verrucomicrobiaceae*, which were even reduced. These results confirm that elevated central levels of αS alone may not lead to early intestinal manifestations in PD (Gorecki et al., 2019). In contrast, induction of PD-related pathology by IP application of LPS in a wt mice led to increased αS expression and accumulation of pathological P-αS specifically in the colon, accompanied by a temporary increased colon permeability. However, nigrostriatal degeneration or other significant pathological changes in the CNS have not been confirmed (Kelly et al., 2014).

### αS-A53T Transgenic Models

Previous studies using experimental animals have confirmed the role of the pathological A53T mutation of αS in the development of PD-related motor and non-motor dysfunctions, such as age-related anxiety and loss of olfaction associated with αS aggregation in the olfactory bulb as well as myenteric plexus and adrenal neurons (Farrell et al., 2014; Kujawska and Jodynis-Liebert, 2018; Taguchi et al., 2020b). Similarly, in PrP-A53T-αS (M83 tg) mice, the age-related significant accumulation of P-αS was detected first in the intestinal nervous system and later in the brain, but in both cases, it preceded the onset of motor symptoms of the disease (Bencsik et al., 2014). The combination of oral paraquat application in M83 tg mice accelerated the onset of αS-associated pathology, with motor dysfunction as well as the presence of P-αS and neuroinflammation in ENS already present in young mice (Naudet et al., 2017). In the context of GIT symptoms, already young M83 tg mice show remarkable signs of gastrointestinal dysfunction, which precede motor abnormalities and CNS pathology. The presence of aggregated forms of αS has been detected in neurons in both myenteric and submucosal plexuses but specifically only in the colon. The problems with intestinal peristalsis are indicated by a slowed transit time through the colon and an abnormal stool. This assertion was supported by electrically evoked contractions of the colon, which showed a reduced motor response in these mice (Rota et al., 2019).

Recently, a new BAC-A53T-αS mouse model with the insertion of two single nucleotide polymorphisms associated with familial PD or with an increased risk of sporadic PD has been published. The expression of pathological forms of αS (truncated, oligomeric and phosphorylated) has been confirmed in areas consistent with clinical findings, including the olfactory bulb, cerebral cortex, striatum and *SNpc*. Among the first symptoms, the authors observed REM sleep disorders, with P-αS present in areas regulating neuronal populations in the lower brainstem such as the sublaterodorsal tegmental nucleus, nuclei in the ventromedial medullary reticular formation, and pedunculopontine nuclei. With the delay, the present hyposmia correlated with the accumulation of P-αS in the olfactory bulb. These manifestations appeared long before the degeneration of dopaminergic neurons in the *SNpc*. However, the authors did not observe any associated motor dysfunction (Taguchi et al., 2020a). Together, these results support the hypothesis that GIT dysfunction and other non-motor symptoms represent an early symptom of αS-mediated pathology without parallel involvement of the CNS (Farrell et al., 2014; Naudet et al., 2017; Rota et al., 2019; Taguchi et al., 2020a).

### Other Genetic Models

Mutations in *phosphatase and tensin homolog*- (PTEN-) *induced novel kinase 1* (PINK1) are associated with early-onset PD. In addition to its role in mitophagy (Narendra et al., 2008), it also plays an important role in adaptive immunity (Matheoud et al., 2016), supporting the hypothesis that autoimmune mechanisms are involved in the etiology of Parkinson’s disease (Matheoud et al., 2019). The deletion of the *pink1* gene in mouse alone does not reflect the characteristic features of the disease, suggesting that the development of the disease symptoms is likely to occur in conjunction with other factors. In this respect, in a recent interesting study, the authors described that intestinal infection of PINK^–/–^ mice with Gram-negative bacteria induces autoimmune mechanisms not only in the periphery but also in the CNS, which are presented by a decrease in the density of dopaminergic axonal varices in the striatum or L-DOPA responsive motor functions. The authors suggest that PINK1 also plays a role in immune system repression and support the gut–brain axis hypothesis of PD-related pathomechanisms (Matheoud et al., 2019).

MitoPark is a chronic, progressive mouse model that recapitulates several key motor (Ekstrand et al., 2007; Li et al., 2013; Ay et al., 2017; Langley et al., 2018) as well as non-motor (Ekstrand and Galter, 2009; Li et al., 2013; Ghaisas et al., 2019) aspects of PD. From the point of view of the GIT itself, among the first non-motor symptoms of PD, decreased motility of the GIT was observed with gradual progression of colon transit times, reduced fecal water content and intestinal inflammation characterized by activation of glial cells in the myenteric plexus. Later, a loss of TH+ neurons was also observed, as well as reduced levels of DA in midbrain as well as in the intestine. Metabolomics analysis also showed slight changes in the composition of the intestinal microbiota or bacterial metabolites (Ghaisas et al., 2019). In this context, Hsieh et al. (2020) observed that long-term administration of probiotics has neuroprotective effects on dopamine neurons and significantly reduced motor dysfunction in MitoPark PD mice (Hsieh et al., 2020). These results suggest the neuroprotective potential of probiotics, probably due to their inhibition of glial cell activation and neuroinflammation, increased butyrate levels, and increased levels of some neurotrophic factors (Fang, 2019; Srivastav et al., 2019). However, the effects and basic mechanisms of such probiotic treatment in PD are unclear (Ghaisas et al., 2019).

### αS Propagation Models

Although αS propagation models are still in development, but due to the shorter onset of the phenotype, their widespread use in terms of experimental models, they represent a promising model in the study of prodromal PD (Kim et al., 2019; Taguchi et al., 2020b). However, it should be borne in mind that different approaches to the purification of αS fibrils, as well as application in different models, can significantly affect the degree of their spread in cells as well as the observed phenotype (Uemura et al., 2018; Kim et al., 2019).

One possibility is the purification of pathological forms of αS from extracts of human brain tissue from patients with dementia with LB (DLB) (Lee et al., 2011) or PD (Holmqvist et al., 2014). Injection of human brain tissue extracts from DLB patients containing stable αS aggregates into the gastric walls of transgenic M83 tg mice resulted in increased deposition and aggregation of αS in myenteric neurons, accompanied by a transiently increased inflammatory response in the GIT. These results indicate that pathological αS aggregates may induce aggregation of endogenous αS in myenteric neurons in M83 tg mice, suggesting the transmission of αS-associated pathology within the ENS (Lee et al., 2011). Application of human brain tissue extracts from PD patients containing various physiological as well as pathological forms of αS to the intestinal wall of rats confirmed retrograde transport of aggregated αS through vagal nerves from the intestine to the brain and that slow and fast components of axonal transport are involved in this process (Holmqvist et al., 2014).

The second, frequently used, the possibility is the preparation of recombinant αS preformed fibrils (PFF). Intramuscular administration of PFF in M83 tg transgenic mice resulted in motor disorders accompanied by the presence of CNS protein inclusions and neuroinflammation. Transection of the sciatic nerve in these mice significantly delayed the onset of CNS pathology as well as motor symptoms themselves, suggesting the involvement of retrograde transport in the induction of αS pathology in the CNS (Sacino et al., 2014). Intraperitoneal or intraglossal application of PFF in M83 tg led to the development of paralysis and the presence of aggregated as well as P-αS in the CNS and spinal cord (Breid et al., 2016). In another study, the authors focused on the propagation of PFFs applied to the olfactory bulb of wild-type (WT) mice. They observed the progression of αS-associated pathology initially manifested by the olfactory disorders, and gradually spread to distant areas of the brain (Rey et al., 2018). Uemura et al. (2018) focused on the propagation of αS-associated pathology mediated by the injection of PFF into the gastric wall of WT or αS-A53T BAC transgenic mice (Uemura et al., 2020). In the case of WT mice, the presence of aggregates of P-αS was observed in the DMV, which, however, did not progress further. Performed vagotomy resulted in a gradual reduction of P-αS aggregates in DMV but did not affect the P-αS-positive aggregates in the myenteric plexus (Uemura et al., 2018). When αS PFF were applied to the gastric wall of αS A53T BAC transgenic mice, they observed a significant increase in αS levels in the brain as well as in stomach compared to WT mice, along with an increased amount of P-αS in DMV. These results indicate, that BAC transgenic expression of αS promoted the propagation of αS-associated pathology in the brainstem, but not subsequent caudo-rostral spread in line with Braak’s hypothesis (Uemura et al., 2020).

## Possible Pathways of PD Pathology Propagation From ENS to CNS

According to Braak’s model, neuropathological changes from GIT are transmitted through anatomical connections between ENS and CNS (Braak et al., 2003, 2006), with the initiation of PD pathogenesis in the ENS and DMV much earlier than *SNpc* pathology. This hypothesis is also supported by the results of biopsies, experimental animals but also with the use of cellular models. For example, in cellular models, the oligomeric αS can be endocytosed by neurons and induce αS aggregation in primary neuronal cultures (Lee et al., 2008; Desplats et al., 2009; Liu et al., 2009). Several either toxin-induced (Pan-Montojo et al., 2012) or PFF-propagation (Holmqvist et al., 2014; Uemura et al., 2018; Kim et al., 2019) mouse models demonstrated vagus-dependent transmission of αS-pathology from peripheral tissues to the CNS followed by the development of motor symptoms. Slight differences in observations in these studies, such as the degree of αS-pathology in the CNS, in some cases its gradual decrease with the inability to lead to the loss of dopaminergic neurons, may also be the result of various PFF purification as well as application protocols used in experiments (Kim et al., 2019). However, important findings were that the hemivagotomy or sympathectomy blocked (Pan-Montojo et al., 2012; Kim et al., 2019), reduced or at least delayed the observed CNS pathology. Similarly, in patients who underwent truncal vagotomy, the risk of PD was significantly reduced (Svensson et al., 2015; Tysnes et al., 2015). All these data suggest that transmission via nervus vagus is one of the possible ways of spreading αS-related PD pathology.

On the other hand, in mice with PFF-induced pathology in the duodenum, the presence of αS inclusion, has been observed in the ENS, intermediolateral spinal cord but also stomach, the dorsal motor nucleus of the vagus nerve, locus coeruleus and even heart. In control animals, these pathological features were not observed. These results open new possibilities for bidirectional αS propagation through the *nervus vagus*: duodenum–brainstem–stomach, which means the possibility of secondary transmission of αS pathology through the anterograde pathway after initial retrograde transmission (Van Den Berge et al., 2019).

An interesting view of the origin and development of PD-associated pathology is provided by the so-called “the threshold hypothesis,” which is based on the parallel degeneration of PNS and CNS (Engelender and Isacson, 2017). According to this theory, almost non-motor symptoms in PD are the result of a “lower functional threshold of enteric neurons,” due to their less-developed network with a smaller number of compensatory mechanisms and connections in the ENS compared to the CNS. As PD-associated motor symptoms occur with the loss of approximately 70% of dopaminergic neurons in the *SNpC*, multiple compensatory mechanisms are thought to exist in the CNS (Marsden, 1982; Hantraye et al., 1992; Wullner et al., 1994; Brownell et al., 1998; Cooper et al., 2009; Mou et al., 2020).

The authors claim that this principle reflects current knowledge of the neurobiology of PD better compared to Braak’s hypothesis. The main counter-arguments are that the only presumed mechanism of selective sensitivity of certain enteric neurons is the less developed compensatory mechanisms in the ENS and also that it does not explain experimental data on αS -associated pathology between ENS and CNS (Mou et al., 2020).

Finally, we must not forget the observed changes in the composition of the intestinal microbiota in PD patients as well as in experimental animals. These changes may affect the local immune response, which leads to disruption of the mucosal barrier of the GIT and subsequent inflammatory reactions. Systemic inflammation as a result of migrated gut bacteria and cytokines may results in an impairment of BBB, progressively to neuroinflammation and through the over-response of the CNS immune system or CNS inflammatory response to neurodegeneration (Maes and Leunis, 2008; Clairembault et al., 2015b; Yang et al., 2017; Morais et al., 2018; Perez-Pardo et al., 2018; Dodiya et al., 2020). However, the role of the intestinal microbiota in the initiation and progression of PD-related pathology is currently unclear, as well as whether these changes in the intestinal microbiome are the cause or consequence of aS-related pathology. In any case, changes in the composition of the intestinal microbiome, as well as gut inflammation, are considered a risk factor in the development of PD (Chapelet et al., 2019).

## Concluding Remarks and Perspectives

The finding that primary PD pathogenesis occurs in peripheral tissues already several years prior the onset of typical motor symptoms suggests that it is extremely important to focus further PD research on early detection of disease, which could bring new treatment options to patients in early stages of PD and improvement of the life quality.

However, despite the incredible efforts and progress in this area, a number of principal questions remain unanswered. One of them poses the question of why, despite the presence of pathological αS forms in peripheral tissues, for example in the appendix (Killinger et al., 2018), there is no pathogenesis of PD in all humans (Mazurskyy and Howitt, 2019).

Moreover, it is currently unknown which GIT tissue would be most suitable for early detection of neuropathological PD changes with respect to minimal invasiveness and patient safety. Therefore, further research into the pathogenesis of PD would need to focus on a more comprehensive examination of tissues, whether obtained from appropriate animal models or biopsy patients obtained both in the pre-motor stage of PD and after the onset of neuropathological changes in the CNS and the development of motor symptoms. These more detailed and, in particular, more comprehensive studies would help several questions to be answered:

(i) in which part of the GIT the pathogenesis of PD occurs
(ii) whether the pathology from that particular tissue progressively spreads to the CNS
(iii) or is first disseminated within the GIT itself
(iv) or the onset is localized across the entire GIT.

**FIGURE 2:**
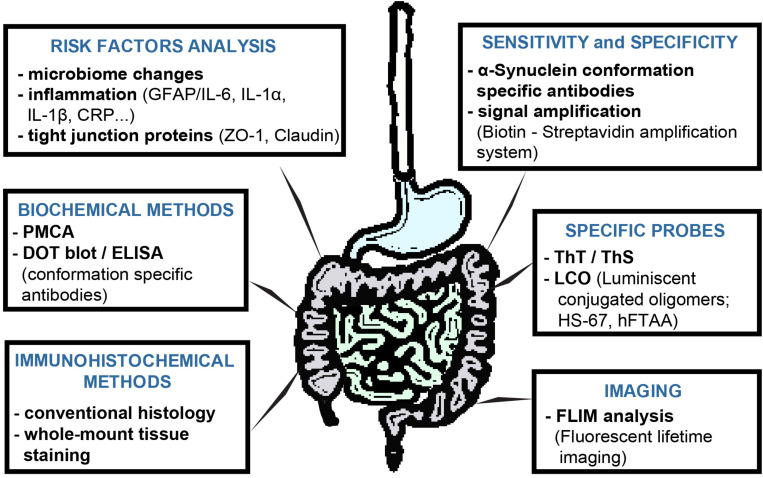
Possibilities to extend of αS-detection methods in GIT tissues of PD patients or animal models. Each method has its limits, so in order to find a reliable method of PD diagnosis, a more comprehensive approach to the analysis of studied tissues is necessary. In contrast to conventional immunohistochemistry, immunohistochemical analysis of whole-mount tissues provides a more comprehensive view of the processes taking place in the studied tissue under. The conformation-specific antibody may significantly improve the ability to detect pathological αS, as the detection of total αS, or its phosphorylated form, does not appear to be sufficient at present. These can be used not only for immunohistochemical or biochemical analyzes and, together with various currently widely available signal amplification systems, they can increase not only the specificity but also the sensitivity of the given methods. PMCA appears to be very versatile and specific in detecting αS pathological forms, as well as in distinguishing individual pathological αS strains. Imaging methods may rely on the continuous development of either fluorescent probes or the development of the methodologies themselves. Luminescent-conjugated oligothiophenes (LCO) represent a new generation of probes with the ability to detect a broader spectrum of protein aggregate proteins, compared to conventional probes. In addition, based on changes in emission spectra, they also provide information on the conformation of the studied aggregated proteins. Fluorescent lifetime imaging (FLIM) reflects the qualitative changes near fluorophore, and therefore with higher sensitivity can detect very small, such as conformational, changes. Although the role of the intestinal microbiome in the PD pathogenesis is currently unclear, analysis of the intestinal microbiota, inflammatory response or permeability of the intestinal barrier can provide a more comprehensive view of the processes taking place in the GIT.

The next limiting step in such studies is the methodology itself. Each method, as we all know very well, has its limitations, advantages, and disadvantages, therefore, it is necessary not only to continually improve and optimize these methods but also to confirm the obtained results with other methodologies. Recent studies suggest that a combination of several methodologies could provide answers to the questions asked (Fenyi et al., 2019) (Figure 2). A large number of studies that we summarized in this review are based on immunohistochemical detection of neuropathological changes in the peripheral tissues of PD patients. In these types of experiments, however, very few studies are concerned with comprehensive research into PD neuropathology, often lacking appropriate control subjects, colocalization of several types of pathological forms of αS, or confirmation of results by biochemical methods. Animal experiments can provide a more comprehensive view, but in these cases, conventional immunohistochemical analysis is often combined only with behavioral tests or basic biochemical methods.

Detection of pathological changes by classical histology with analysis and staining of microsections obtained from patient biopsies has, similar to other methods, its limitations. These arise mainly from the nature of the methodology where the tissue is not analyzed as a whole, which can lead to the loss of important information within individual micro-sections. A significant advantage of this methodology, however, is the minimal invasiveness in the removal of biological material from a living patient. Unlike micro-section analysis, the wholemount tissue analysis approach can visualize and analyze innervation from a larger area of the GIT, at a greater depth that can provide a more comprehensive view of the processes taking place in a given tissue (Wang et al., 2012; Harsanyiova et al., 2019). Increasing the sensitivity of immunohistochemical staining, for example by the amplification method (Harsanyiova et al., 2019), could help detect even minimal levels of various pathological forms of αS. The availability of antibodies against a wider spectrum of pathological forms of αS makes it possible to extend the possibilities of immunohistochemical staining and may suggest a time course of the disease.

It is believed that the toxicity of oligomeric forms of αS is higher than insoluble fibrils. Therefore, it is the oligomeric forms that are considered to be initiators of neurodegeneration not only in PD but also in other neurodegenerative diseases (Glabe and Kayed, 2006; Benilova et al., 2012; Mou et al., 2020). Conformational-specific antibodies may be useful in detecting the initiation stages of αS-associated pathology in GIT tissues, not only by immunohistochemical staining of tissues but in some cases also in biochemical analyzes such as, e.g., dot blot or ELISA. The PMCA assay utilizes the functional properties of oligomers to inoculate polymerization of monomeric protein. Its versatility and high specificity are confirmed by studies where PMCA has been used to detect the pathological forms of αS in colony biopsies (Fenyi et al., 2019), or cerebrospinal fluid (Shahnawaz et al., 2017; Kang et al., 2019). PMCA is also able to distinguish different conformational strains of αS that are involved in the development of various synucleinopathies, as confirmed by analysis of samples from patients with PD multiple systemic atrophy (MSA), with an overall sensitivity of up to 95.4% (Shahnawaz et al., 2020).

Furthermore, using more sophisticated FLIM-based detection methods that analyze the lifetime of fluorescence emitted by endogenous or exogenous fluorophores can help resolve the onset and progression of PD pathogenesis at the very early stages of the disease. The lifetime of fluorescence emitted by a fluorophore is affected by the environment of a given fluorophore, which allows the detection of even very small changes (e.g., conformational changes of αS) with higher sensitivity compared to immunohistochemical or biochemical methods.

Several fluorescent probes used as a “gold standard” (Thioflavin S and Thioflavin T) in the detection of aggregated proteins associated with neurodegenerative diseases have been successfully used in FLIM analysis not only in recombinant systems (Plotegher et al., 2015) but also in cellular models (Pokusa and Kralova Trancikova, 2018). Furthermore, FLIM analysis using luminescent conjugated oligothiophenes (LCOs) was able to distinguish αS-positive inclusions in the brain sections of the brain of PD patients and patients with MSA (Klingstedt et al., 2019 #174). LCOs are, in comparison with classical fluorescent probes, such as Thioflavins or Congo red, able to detect a wider range of protein aggregates. Due to their flexible thiophene backbone, the emitted light depends on their binding to proteins in different conformations. In addition, differences in the emission spectra of these LCOs reveals different conformations of αS strains in patients with PD and MSA (Klingstedt et al., 2011, 2019).

## Author Contributions

JH focused on the detection of pathological processes in the tissues of the GIT in animal models. She also specializes in the optimization of detection methods such as classical histology, immunostaining of wholemount tissues, amplification of the detection signal and further analysis by confocal and multiphoton microscopy. TB focused on the clinical part of the manuscript, where he can best use his MD education and experience. AK focused on animal and genetic models of Parkinson’s disease, where she can best use her long-term experience in this field. Also, she focused on the possibilities of improving the detection of PD-related pathological changes in the tissues of the GIT, in particular confocal microscopy and FLIM analysis. All authors contributed to the article and approved the submitted version.

## Conflict of Interest

The authors declare that the research was conducted in the absence of any commercial or financial relationships that could be construed as a potential conflict of interest.
